# The potentials for incidental vocabulary acquisition from listening to computer science academic lectures: a higher education corpus-based case study from Macau

**DOI:** 10.3389/fpsyg.2023.1219159

**Published:** 2023-07-26

**Authors:** Barry Lee Reynolds, Xiaowen (Serina) Xie, Quy Huynh Phu Pham

**Affiliations:** ^1^Faculty of Education, University of Macau, Macao, Macao SAR, China; ^2^Centre for Cognitive and Brain Sciences, University of Macau, Macao, Macao SAR, China; ^3^Moray House School of Education and Sport, University of Edinburgh, Edinburgh, United Kingdom; ^4^School of Languages and Cultures, The University of Queensland, Brisbane, QLD, Australia

**Keywords:** EMI, incidental vocabulary acquisition, lectures, computer science, case study, corpora

## Abstract

**Introduction:**

Universities in non-Anglophone countries are increasingly implementing English as the medium of instruction (EMI) lectures. There seems to be an assumption that students’ performance on standardized English examinations can be equated with the lexical knowledge needed to comprehend EMI lectures regardless of discipline. For unknown words students encounter, it is assumed that they can be *picked up* through listening to these lectures. This potential for students to acquire unknown words incidentally while listening to these lectures has yet to be fully explored.

**Methods:**

This study addresses the potential of students incidentally acquiring vocabulary from listening to EMI lectures through corpus analyses of computer science lectures at one public university in Macau. Taking into consideration frequency, range, and lecturer explanation, corpus analyses of the transcripts of 28 computer science lectures (40 h 36 min) were conducted to determine the lexical knowledge needed for students to comprehend the lectures. The potential number of words these students could acquire through listening to the lectures was also uncovered through further analyses.

**Results:**

Results showed that L2 students need to have receptive knowledge of the most frequent 3,000 word families plus proper nouns and marginal words to reach beyond 95% lexical coverage. To reach 98% lexical coverage, 5,000 word families are needed. Considering frequency, range, and teacher explanation, we concluded that 30 new words could reasonably be incidentally acquired after listening to the 28 lectures.

**Discussion:**

These results indicate a need for EMI lecturers to consider the lexical knowledge of students and whether additional pedagogical techniques (i.e., vocabulary explanation) should be employed in content classrooms when lectures are delivered in English, especially for specialized fields such as computer science. Our results also draw attention to the importance of field specific vocabulary and the potential pitfalls of using blanket English language admissions criteria when admitting students to different academic programs.

## 1. Introduction

In recent years, English as the medium of instruction (EMI) has gained attention and popularity worldwide, with voices being heard from micro, meso, and macro levels in contexts like Hong Kong and Mainland China, among others (Xie and Peng, 2023). Similarly, EMI has become fully implemented in Macau’s higher education, whereas the potential for students’ vocabulary learning from EMI lectures remains unclear. In addition to focusing on localization of education through heavy recruitment of local Macau residents, there has been an increasing number of enrolments in EMI programs by many students from mainland China and overseas (Education and Youth Development Bureau, 2022). EMI has been promoted for both English language teaching and content teaching in Macau’s higher education to address the institutional need for internationalization of higher education. For instance, Macau University of Science and Technology (2022) specifically points out that offering EMI programs develops bilingual students.

To recruit students for these EMI programs, Macau higher education institutions use standardized English examinations as gatekeepers, some of which further categorize prospective students as *qualified for entry to EMI programs* and *in need for pre-sessional English courses*. For instance, the University of Macau and the Macau University of Science and Technology both use IELTS band 6.0/9.0 and TOEFL 80/120 as the benchmarks for this purpose when enrolling students for their EMI undergraduate programs (Macau University of Science and Technology, 2022; University of Macau, 2022). Pre-sessional English for academic purposes (EAP) courses are offered in universities that admit students in need of help to meet the entry-level English language requirements necessary for studying in EMI undergraduate programs. Specifically, the EAP courses are offered to assist students’ English proficiency development, contributing to getting students *ready* for learning content through EMI, similar to what McKinley et al. (2021) found in mainland China.

It is uncertain how these benchmarks may represent the *readiness* of students for EMI study. Although these benchmarks may be useful for student admission, they may not indicate students’ repertoire of specialized vocabulary. It is also unclear what percentage of the words (i.e., lexical coverage) students should understand before they can reasonably be expected to comprehend and learn content knowledge from EMI lectures. Instead, higher education institutions have circumvented this issue by assuming that a received number of English language instruction hours can be used to infer English language proficiency based on the Common European Framework of Reference for Languages (CEFR). After a student enters university, a simple calculation is made to specify the number of English language instruction hours necessary for the student to progress from the *in need for pre-sessional English courses* status to *qualified for entry to EMI programs* status.

While there might very well be a correlation between CEFR and hours of language instruction, it is problematic not to consider students’ vocabulary knowledge when discussing readiness for attending EMI lectures. Researchers have argued that vocabulary knowledge is highly correlated with students’ EMI learning outcomes (Uchihara and Harada, 2018). Macaro (2022) emphasized students’ often-voiced claim that vocabulary is a main challenge in EMI programs and Tsou (2021) further suggested that some of the standardized English examinations used as gatekeepers for students’ enrollment in tertiary EMI programs is normally based on the testing of reading and listening comprehension in *general academic* English. Thus, these standardized exams may fail to provide an evaluation for students’ command of specialized vocabulary that is demanded for their listening comprehension while listening to the EMI lectures. It thus appears that EMI policies may tacitly assume students can acquire other necessary vocabulary incidentally through listening to EMI lectures even if they begin their studies with inadequate vocabulary sizes.

It is important to consider whether the vocabulary learners are exposed to through course materials, whether in EMI programs or English language courses, are at the appropriate level for the students to understand (Nation, 2006, 2022). However, both English language textbooks and EMI context textbooks (e.g., science) have been shown to be flooded with words that would be unknown to students reading these texts; these unknown words may not necessarily be useful for understanding, thereby hindering their comprehension of the content of those materials (Hu et al., 2021; Nguyen, 2021). These textbook analysis studies aimed at showing how the vocabulary knowledge of most students will be incongruent with the vocabulary used in the texts. By contrast, more recent quasi-experimental research (Song and Reynolds, 2022a,b) has found that after taking into consideration topic familiarity and second language (L2) vocabulary size, as lexical coverage (i.e., the words known by readers in a text) increases, comprehension of narrative and expository texts generally increases.

It is thus reasonable to expect that low lexical coverage of aural input may also decrease the chances of incidental vocabulary learning through listening to EMI lectures as comprehension of the contexts surrounding unknown words is a prerequisite for incidental acquisition (Mondria and Wit-de Boer, 1991; Van den Broek et al., 2018). Likewise, there is some indication that increasing lexical coverage of oral input, for example when watching television programs, positively affects comprehension and incidental vocabulary learning (Rodgers, 2013). However, given the absence of vocabulary research in EMI classrooms, the potentials for incidental vocabulary acquisition through listening to EMI lectures remain unclear. As policymakers and administrators in Macau support the implementation of EMI in Macau’s higher education, it is urgent to understand which words can potentially be new to students.

EMI education has been introduced to Macau from the macro- and meso- levels, but there is a lack of research to examine incidental vocabulary acquisition from EMI lectures. Like other governments promoting EMI policy and implementation for the benefits of internationalization and commercialization of higher education (Bowles and Murphy, 2020), the Macau SAR government and the higher education institutions in Macau are no exception (Education and Youth Development Bureau, 2021; Macau University of Science and Technology, 2022; Macao Institute for Tourism Studies, 2023). However, it should not be forgotten that these movements may cause potential issues, uncertainties, and challenges to the EMI course lecturers and students at the micro-level (Ma et al., 2022). Macaro (2022) emphasized the need for lecturers to explore both vocabulary coverage and students’ learning strategies for successful comprehension of EMI lectures. After all, practitioners and students are the critical *agents* (Priestley et al., 2015; Nagai et al., 2020) that make internationalization and commercialization of higher education possible.

In this case study we investigated the potential for students to incidentally acquire vocabulary by listening to EMI lectures. Subsequently, this study sheds light on EMI curriculum development, pedagogical innovation, interdisciplinary collaboration, and EMI teacher professional development. With these contributions, the study is highly beneficial for a wide range of stakeholders implementing EMI in Macau and similar contexts.

In the sections that follow, we first critically review the relevant EMI and incidental vocabulary acquisition literature, and then provide the problem statement, research aims, and research questions. Details of the research design then follow. Results from the corpus analyses and the discussion of these results are then presented before moving on to the conclusion and implications. Finally, the limitations and future directions are reported.

## 2. Literature review

### 2.1. Potentials for incidental vocabulary acquisition through listening to EMI lectures

Much of the EMI research has focused on issues related to students’ gains in content knowledge or general language proficiency but not vocabulary development (e.g., Galloway et al., 2020). While there have been claims that vocabulary knowledge is the key to language learning and use (Alijanian et al., 2019), the analysis of EMI syllabi and interviews with students enrolled in EMI courses found vocabulary knowledge development was prioritized less than content knowledge (Fang and Xie, 2019). Although EMI syllabi do not often include explicit language learning goals, these learning goals are “often assumed by the policy makers” (Galloway and Rose, 2021, p. 34). Whether stated directly or not, most EMI lecturers would agree that vocabulary does serve as a vehicle for students to comprehend, acquire, apply, and complete content assessments. From the students’ perspective, vocabulary knowledge is a significant driving force for them to be able to further develop English proficiency through enrolment in EMI courses (Galloway et al., 2017). Thus, the expectation of language learning, especially vocabulary acquisition to occur incidentally through content learning in EMI higher education, remains implicitly embedded and prioritized in the practice of EMI.

Instead of investigating the potential value of and suggesting practical proposals for using EMI lectures to enhance students’ vocabulary knowledge, researchers have called for offering various linguistic support to students (Wang et al., 2018) and professional development support to EMI lecturers (Macaro et al., 2021). Research has also suggested encouraging cooperation between content and language teachers (Hu and Gao, 2018). However, content teachers that deliver EMI lectures may not perceive themselves as language teachers and therefore may believe that EAP teachers should assist students with linguistic issues (Galloway and Ruegg, 2020). Nevertheless, assessments in EMI courses are often delivered in English, with unstated marking criteria that may sometimes prioritize the usage of English vocabulary and grammar, as some EMI lecturers have been shown to comment on students’ English grammar and vocabulary use (Fang and Xie, 2019).

Although the assumption that EMI can enhance students’ English learning is often embedded in EMI course curricula, less is known about the actual potential for incidental vocabulary acquisition through attending EMI programs. While there are some studies that have been conducted on the potentials of incidental vocabulary acquisition through the listening to teacher talk or limited English lecturing (e.g., Horst, 2010; Jin and Webb, 2020), these studies have not adequately addressed whether or not it is feasible for students to acquire vocabulary through enrolment in EMI programs of study. One exception was a case study by Reynolds et al. (2022), that examined how listening to English language teaching EMI lectures for a single course could result in a modest amount of incidental learning of vocabulary. However, more understanding of the potentials for incidental vocabulary acquisition in other disciplines, such as computer science, is needed, as discipline differences in EMI programs (Peng and Xie, 2021) may pose varied vocabulary demands and target learning outcome that are linked to students’ command of specialized vocabulary. Hence, this case study aims to provide discipline-specific and context-specific knowledge for future EMI provision in Macau and similar contexts that offer EMI to facilitate students’ incidental vocabulary acquisition through engagement with discipline-specific content.

### 2.2. Lexical profiling of academic lectures

Lexical coverage is “the degree to which words in input are known by readers and listeners” (Webb, 2021a, p. 278). For instance, if a student knows 90 out of 100 running words, then his or her lexical coverage is 90%. In the research literature, lexical coverage in combination with several other variables has been deemed indicative of comprehension. The lexical coverage figures of 95% and 98% have been used in a number of lexical profiling spoken academic discourse studies (e.g., Dang and Webb, 2014; Dang, 2022; Reynolds et al., 2022), and the use of these figures as conventions in corpus studies has arrived from an increasing number of studies advocating 95% or 98% lexical coverage as an adequate coverage of lexis allowing for reasonable understanding of language input (e.g., Hu and Nation, 2000; Laufer, 2020; Song and Reynolds, 2022a,b; *inter alia*). Accordingly, the present study builds upon these previous studies’ findings and discussions (e.g., Dang and Webb, 2014; Dang, 2022; Reynolds et al., 2022) regarding lexical coverage.

Academic lectures given in different disciplines may require different lexical coverage for students. Dang and Webb (2014) examined the lexical demands of English speeches in four academic disciplines, including Arts and Humanities, Life and Medical Sciences, Physical Sciences, and Social Sciences. They found that given a vocabulary size consisting of the most frequent 4,000 word families and proper nouns as well as marginal words, 96% coverage would be achieved. Additionally, knowing the most frequent 8,000 word families, proper nouns as well as marginal words would contribute to 98% coverage. Later, after examining lexical demands in tertiary EMI lectures, non-EMI courses, and open-access non-EMI courses, Dang (2022) found that in comparison, non-EMI lectures were more lexically demanding than EMI lectures. Although EMI lectures required students to understand the most frequent 7,000 word families, non-EMI lectures required students to have obtained knowledge of the most frequent 9,000 word families to achieve 98% coverage. As for the open-access non-EMI lectures, students needed to understand the most frequent 8,000 word families to achieve the same 98% coverage. Recently, Reynolds et al. (2022) found that in order to reach 98% coverage of the EMI lectures in an *Introduction to English Language Teaching* course offered in a Macau higher education institution, students needed a vocabulary size of the most frequent 4,000 word families, proper nouns, and marginal words. However, there are several disciplines, such as computer science investigated in the present study, that have yet to be examined.

### 2.3. Factors contributing to incidental vocabulary acquisition

There are several different factors that can potentially moderate the incidental acquisition of vocabulary encountered through language input such as what occurs in EMI lectures. Given the scope of our study, we will only focus on the effect of frequency, range, and explanation, since these three factors have been extensively discussed in the scholarship on incidental vocabulary learning and likely to have marked effects on incidental learning outcomes.

#### 2.3.1. Frequency

Frequency often refers to the number of encounters with or the occurrences of words targeted for learning (Reynolds and Wible, 2014). A substantial amount of incidental vocabulary acquisition research has been conducted examining how frequency affects word learning, finding a positive effect (e.g., van Zeeland and Schmitt, 2013; Chen and Teng, 2017). When calculating the recurrence of words within language input, researchers have the option of counting only exact word forms (excluding inflectional and derivational variants), lemmas (including inflectional variants), or word families (including inflectional and derivational variants) (Reynolds and Wible, 2014). While different views have been given on which counting unit should be used (e.g., Webb, 2021b), researchers should clearly indicate which unit they have used for calculating frequency and justify that decision when reporting their research methods (Reynolds and Wible, 2014).

van Zeeland and Schmitt (2013) investigated the effect of frequency (3, 7, 11, and 15 encounters) on three dimensions of vocabulary knowledge, including form, grammar, and meaning. They maintained that at least 15 encounters are required for incidental learning of form, grammar, and meaning through listening. Chen and Teng (2017) examined the frequency (1, 5, and 10 encounters) effect on vocabulary development, i.e., form and meaning, through reading and listening. They revealed that it is extremely difficult for incidental learning to occur in a listening-only mode if students only hear the word 1–5 times. In their view, students need to hear the words at least 10 times for incidental vocabulary acquisition. However, recently, Jin and Webb (2020) concluded that frequency, ranging from 3 to 10 encounters, did not significantly contribute to vocabulary learning or retention, a somewhat contradictory finding. They suggested that the insignificant contribution of frequency might have been caused by the limited encounters with target words and the greater influence of the teacher’s first language (L1) translations. Clearly, there is no specific frequency threshold at which students can successfully acquire words incidentally from listening. However, it is generally agreed upon that the more encounters (i.e., higher frequency), the more likely learners can incidentally acquire the unknown words encountered through language input (Reynolds and Wible, 2014). By contrast, if words occur too infrequently, it would be challenging, if not impossible, for students to acquire them incidentally.

#### 2.3.2. Range

Range is defined as “the number of texts in which a given word appears in a corpus” (Hashimoto and Egbert, 2019, p. 8). Unlike frequency, range provides information on how widely a word spreads across multiple texts in a corpus. For example, if a word has a range value of five, it means that this word occurs at least once in five different documents of a given corpus. Given the incremental nature of incidental vocabulary learning (Thomas, 2020), range can potentially affect the incidental acquisition of novel words in the sense that it increases students’ exposure to these words (Hashimoto and Egbert, 2019; Rodgers and Webb, 2019). In psycholinguistics research, the term contextual diversity has been used instead of range (Caldwell-Harris, 2021). This construct has been investigated alongside other related measures such as contextual distinctiveness to determine whether it can explain more of the variance in (incidental) word learning than frequency of exposure (e.g., Rosa et al., 2022). While we can see the benefits of a study that examines contextual diversity alongside frequency, range was examined in the present case study as we felt it is a more straightforward variable easily interpreted and discussed alongside teacher explanation.

In recent years, several studies have been conducted to examine the relationship between range and incidental vocabulary learning (e.g., Webb and Chang, 2015; Rodgers and Webb, 2019; Reynolds and Ding, 2022). Webb and Chang (2015) found no relationship between range and incidental acquisition of new vocabulary. However, it should be noted that most of the target words (i.e., 73 out of 100) in Webb and Chang’s (2015) study appeared in only one text. An alternative interpretation of this finding could be that words of lower range might not result in incidental vocabulary learning. Rodgers and Webb (2019) examined the effect of range on the incidental learning of new words through watching 10 episodes of a television program. They selected 60 words with different range values. It was shown that range had a small negative effect on incidental vocabulary learning, meaning that the more episodes appeared in, the less likely participants learned them. Nevertheless, like the target words in Webb and Chang (2015), the target words in Rodgers and Webb (2019) were also restricted in their range of encounters. Specifically, out of the 60 target words, 48 words occurred in 1 to 5 episodes, and only 12 words occurred in 6 to 10 episodes. In a more recent study conducted by Reynolds and Ding (2022), range was found to correlate significantly with the acquisition of productive word meaning. A closer look at the target words in Reynolds and Ding (2022) reveals that most of the target words had a wider range, compared to those in previous studies. Specifically, out of the 50 target words, only 18 words had a range value from 1 to 5, and the other 32 words had a range value from 6 to 21. Taken together, results from the previous studies suggest that words of a lower range (from 1 to 5) would perhaps reduce the likelihood of new words being incidentally acquired.

#### 2.3.3. Explanation

In addition to the existing examination of frequency and range as factors that affect incidental vocabulary acquisition, explanation has been identified as a useful strategy for students’ vocabulary development. Previously, English language teaching studies have examined the influence of explanation for incidental vocabulary acquisition through listening to teacher talk, but not in EMI settings. Yang and Sun (2013) concluded in their study that English as a foreign language learners could incidentally acquire L2 vocabulary through a single viewing of online lectures. They suggested that the level of explicit and clear explanations associated with verbal and non-verbal elaboration on vocabulary is a significant predictor of the success of incidental vocabulary acquisition. Tian and Macaro (2012) found lexical Focus-on-Form instruction had a significant influence on L2 vocabulary learning. Similarly, Webb and Nation (2017) suggested that providing explicit explanations of words can enhance incidental vocabulary acquisition in L2 classrooms. Nevertheless, other than using L2 only in explanation, Zhao and Macaro (2016) suggested that the use of L1 in explanation can have a more significant impact on incidental vocabulary acquisition. Although Jin and Webb (2020) also found that explanation and L1 translation in teacher talk could have significant influence on students’ likelihood of incidental vocabulary acquisition, they suggested that future studies should involve a larger amount of teacher talk in other contexts to promote generalizability of research findings in the area.

Recently, Dang et al. (2022) conducted an experimental study to investigate the effect of input modes, frequency, type of vocabulary, and elaboration on Chinese EAP students’ incidental vocabulary learning. After introducing five modes of input for an open access academic lecture to their experimental groups, they found that neither verbal elaboration nor non-verbal elaboration could affect students’ incidental vocabulary acquisition. Dang et al. (2022) suggested that their results might be different from previous studies, such as Yang and Sun (2013), because of the longer academic lecture (50 min) used. However, it should be noted that Dang et al. (2022) was not based in EMI classrooms, and they only included one lecture in the study. It is still necessary to consider explanation as a factor in students’ incidental vocabulary acquisition through listening to a series of lectures in EMI classrooms. Thus, although Dang et al. (2022) indicated that explanation might not significantly influence students’ incidental vocabulary acquisition, many researchers have suggested that explanation can be useful for students’ vocabulary gains (e.g., Tian and Macaro, 2012; Yang and Sun, 2013; Zhao and Macaro, 2016; Webb and Nation, 2017; Jin and Webb, 2020). Accordingly, we assume that teacher explanation during EMI lecturing can help induce incidental vocabulary learning.

### 2.4. Problem statement, research aims, and research questions

Previous studies have investigated the influence of frequency, range, and explanation on incidental vocabulary acquisition in English language teaching classrooms, but not in EMI classrooms where the focus is content learning and L1 is not utilized in explanations. Hence, this study provides a clearer picture of how likely vocabulary can be incidentally acquired from listening to EMI lectures by examining a combination of factors (i.e., frequency, range, and the utilization of explanation) in Macau’s tertiary EMI classrooms.

Studies unpacking the potential for incidental vocabulary acquisition through listening to academic EMI lectures in the Macau context are lacking in the extant literature. Previous studies have investigated the possibilities and outcomes for students’ vocabulary learning within EMI higher education in Mainland China (Zhang and Pladevall-Ballester, 2021) and EMI secondary schools in Hong Kong SAR and South Korea (Lo and Murphy, 2010; Hong and Basturkmen, 2020). Although Reynolds et al. (2022) recently examined the potential for incidental vocabulary acquisition through listening to EMI lectures in Macau, their study only involved one academic discipline. The present study expands this area of research to the discipline of computer science in the context of Macau.

This study aims to assist policymakers and administrators from both the meso- and macro-levels in understanding the potentials of EMI lectures for promoting incidental vocabulary acquisition. The findings of this study also have the potential to inform EAP program vocabulary teaching practices. This study also serves as a basis for teacher educators to understand EMI lecturers’ urgent needs for pedagogical strategy enhancement in Macau’s higher education. The pedagogical implications of this study can contribute to other similar contexts by shedding light on the vocabulary needs of students enrolled in EMI programs.

To understand the potentials for incidental vocabulary acquisition from listening to EMI computer science lectures in Macau, the present case study aimed to answer the following research questions:

(1) What is the lexical coverage of the EMI computer science lectures?
(2) Which words did the teacher use that were likely to have been new to the students?
(3) What was the likelihood of the new words being incidentally acquired through listening to the lectures?

## 3. Materials and methods

The method used received ethical review and approval by the Sub-Panel on Social Science and Humanities Research at the University of Macau (SSHRE22-APP014-FED).

### 3.1. Lectures

The lectures collected for the current case study focused on a variety of topics in computer science (e.g., data storage and manipulation, algorithms, programming languages, data structures, artificial intelligence, and computer science as a discipline). Due to the limited access to EMI lectures, we used convenience sampling for the selection of EMI lectures for this case study. The course, *Introduction to Computer Science*, is aimed at undergraduate freshmen (year 1) students. All the lectures were delivered online using Zoom software often accompanied with slide presentations. The professor provided the researchers with access to download the lecture recordings and corresponding auto transcribed transcriptions from the Zoom server. In the lectures, there was minimal interaction between the professor and students. Most of the interaction occurred when the professor asked questions and corrected coding exercises. The duration of all the lectures is 40 h 36 min.

### 3.2. Corpus analyses

In answering RQ1, twenty-eight online lectures were initially auto transcribed by Zoom software (Zoom Video Communications, 2022). Following this was the meticulous examination of the transcribed lectures to amend misspelled words produced by the transcription program. In the current study, Nation’s (2017) British National Corpus (BNC)/Corpus of Contemporary American English (COCA) twenty-five 1,000-word family lists were utilized in conjunction with the four lists of proper nouns, marginal words, compounds, and acronyms. Words that were not found in the wordlists were classified as off-list words. To adhere to the spellings used in the wordlists, contractions (e.g., *doesn’t* or *isn’t*) were converted into their full form, and hyphenated words (e.g., *computer-aided* or *high-tech*) were dehyphenated. The data cleaning process resulted in a corpus of 264,400 tokens (i.e., the occurrence of a word) and 4,950 types (i.e., the unique occurrence of a word regardless of its frequency). The average speech rate was 108 tokens per minute, which is considered moderately slow for academic lectures (Tauroza and Allison, 1990).

Upon the completion of the data cleaning process, the lectures were converted into text files and processed by means of AntWordProfiler (Anthony, 2022) to assess the lexical demand of the lectures. AntWordProfiler has been proven useful to investigate the lexical profile and complexity of text (e.g., Aldohon, 2018; Moser, 2021). In the first round of the data analysis, new proper nouns (e.g., *Spotify* and *Bluetooth*) and marginal words (e.g., *wala* and *mou*) were identified. In our study, proper nouns and marginal words were included in the analysis of lexical coverage on the assumption that students are likely to recognize them in academic speech (e.g., Nation, 2006; Dang, 2020). Thus, newly-identified proper nouns and marginal words were put back into the wordlists. In the case of acronyms, we determined through discussion with the course professor whether they were used like proper nouns (e.g., *PHP*), and if so, would be treated as proper nouns. Meanwhile, we found that eleven proper nouns should be classified as acronyms based on the lectures’ context. Therefore, these proper nouns were treated as acronyms in the current study. Finally, in addition to the calculation of lexical demand of the entire corpus, the lexical demand of individual lectures was also investigated to paint a more comprehensive picture of the lexical demand across all of the lectures.

In answering RQ2, we relied on the English proficiency requirements for students to enroll in EMI programs at the university where the lectures took place. Specifically, these students are expected to achieve a minimum score of 79 out of 120 for TOEFL-iBT or 6.0 out of 9.0 for IELTS (University of Macau, 2022), which is equivalent to the B2 level on the CEFR scale (see ETS, 2022; IELTS, 2022 for official score conversion^1^). According to CEFR (Council of Europe, 2022), language proficiencies are categorized into six levels: A1 (the lowest level), A2, B1, B2, C1, and C2 (the most advanced level). On the CEFR scale, B2-level students are considered as independent language users who are able to comprehend the main ideas of texts across both concrete and abstract topics and interact with native speakers without much difficulty.

Vocabulary size required for the B2 level has been extensively discussed in prior scholarship. For instance, Milton (2009) used the Swansea Levels Test [designed by Meara and Milton (2003)] to map vocabulary size onto the CEFR levels. It was found that B2-level students would probably know the most frequent 3,250 to 3,750 words in English. Huhta et al. (2011) adopted Nation’s (1990) Vocabulary Levels Test (VLT) to examine vocabulary size in accordance with CEFR levels. They showed that knowledge of the most frequent 5,000 word families can differentiate between B2 and C1 levels. In other words, B2-level students would probably know the most frequent 4,000 words, but not those in the 5,000 + frequency lists. Paul Nation (personal communication, 19 June 2023) suggested that B2-level students might know around 4,000 words, of which are 2,000 to 3,000 high frequency words and 1,000 to 2,000 relevant technical vocabulary. Taken together, it seems that students at B2 level on the CEFR would probably know about 4,000 English words.

It should be noted that previous studies employ different vocabulary level tests to measure vocabulary size alignment with CEFR B2 (e.g., Milton, 2009; Huhta et al., 2011). Therefore, to maximize the chance that words extracted from the 5,000 + frequency level list and off-list words are unknown to students attending the lectures, we invited both the course professor and a language instructor at the University of Macau to provide their opinion on the difficulty level of the extracted words. The motivation behind this decision was due to the fact that many students at the university would have to participate in pre-sessional English courses prior to their official enrollment in the EMI programs. The inclusion of both the course professor and language instructor helps provide different perspectives on the difficulty level of the words (e.g., Ackermann and Chen, 2013), consequently painting a more reliable picture of what words are potentially unknown to B2-level students.

Regarding the raters’ backgrounds, the course professor has 25 years of teaching experience. He speaks English as a second language (CEFR C2 + proficient user) and Portuguese as a first language. In addition, his listening and speaking of Cantonese is at the independent user level (CEFR B1). The language expert has 17 years of English language teaching experience and holds a Doctorate in Applied Linguistics, Master’s in TESOL, and a postgraduate diploma in Second Language Teaching. He speaks English as a first language. In terms of the word rating process, it was performed after the completion of the course. Both raters were instructed to rate the difficulty level of the words on a scale from 1 to 4 (i.e., 1 means the word is definitely unknown to students, 2 means the word may be unknown to students, 3 means the word may be known to students, and 4 means the word is definitely known to students). Raters were instructed to take into consideration various important factors while performing their rating, such as students’ English language proficiency (i.e., at least B2 level), students’ years of enrollment (i.e., first year), students’ majors (i.e., computer science), the learning context (i.e., the University of Macau), and the EMI nature of the course. Raters also received a detailed description of the B2 level to improve their understanding of the lexical demands expected at this level. Finally, given the focus of the present study is on the number of words potentially new to students, words rated 4 by both raters would be excluded from future analysis.

In answering RQ3, given conflicting evidence on the frequency effect on incidental vocabulary acquisition, we classified words in the 5,000 frequency level and beyond into four frequency bands: Words that occurred (1) 1–4 times, (2) 5–9 times, (3) 10–14 times, and (4) at least 15 times. In doing so, we aim to paint a more comprehensive picture of the distribution of the new words at specific frequency levels. Furthermore, as discussed in the previous section, students attending the course were believed to be at least at the B2 CEFR level, and words in the 5,000 frequency level and beyond might be unknown to them. By looking at the frequency of occurrence of these words, we can estimate how many new words students can acquire incidentally through listening to academic lectures. Finally, to count the frequency of occurrence of a word, we adopted word family^2^ as the counting unit since it is regarded as a suitable approach in the case of receptive knowledge (e.g., listening comprehension) (Nation and Webb, 2011; Reynolds and Wible, 2014; Vilkaitė-Lozdienė and Schmitt, 2020). In other words, all occurrences of each variant form of a word were counted. For example, if *comfort* occurred 4 times, *comfortable* 1 time; *comforting* 2 times; *comfortably* 3 times, the cumulative frequency of occurrence of the word *comfort* would be 10 times.

## 4. Results

The descriptive statistics show that most of the words in the present corpus belong to the first 1,000-word family list. Specifically, 230,238 out of 264,400 word tokens, 1,760 out of 4,950 word types, and 839 out of 2,913 word families come from the most common 1,000 word families (see Table 1). Given that the corpus data were compiled based on spoken language, the result was unsurprising.

**TABLE 1:** Tokens, types, and word families at each word level for the entire present corpus.

Word list	Tokens		Types		Word families	
	Raw	%	Raw	%	Raw	%
1,000	230,238	87.08	1,760	35.56	839	28.8
2,000	14,743	5.58	1,067	21.56	535	18.37
3,000	8,128	3.07	804	16.24	463	15.89
4,000	2,062	0.78	301	6.08	210	7.21
5,000	1,279	0.48	152	3.07	107	3.67
6,000	1,020	0.39	86	1.74	64	2.2
7,000	449	0.17	63	1.27	47	1.61
8,000	443	0.17	58	1.17	44	1.51
9,000	239	0.09	28	0.57	20	0.69
10,000	252	0.1	30	0.61	20	0.69
11,000	341	0.13	19	0.38	16	0.55
12,000	110	0.04	10	0.2	8	0.27
13,000	114	0.04	10	0.2	9	0.31
14,000	81	0.03	12	0.24	11	0.38
15,000	9	0	5	0.1	5	0.17
16,000	51	0.02	10	0.2	8	0.27
17,000	40	0.02	10	0.2	6	0.21
18,000	143	0.05	7	0.14	6	0.21
19,000	12	0	2	0.04	2	0.07
20,000	10	0	4	0.08	3	0.1
21,000	6	0	2	0.04	2	0.07
22,000	23	0.01	1	0.02	1	0.03
23,000	23	0.01	3	0.06	3	0.1
24,000	0	0	0	0	0	0
25,000	5	0	3	0.06	3	0.1
Proper nouns	1,478	0.56	202	4.08	199	6.83
Marginal words	1,702	0.64	27	0.55	25	0.86
Compounds	473	0.18	80	1.62	66	2.27
Acronyms	427	0.16	47	0.95	44	1.51
Off-list words	499	0.19	147	2.97	147	5.05
Total	264,400		4,950		2,913	

In answering RQ1, it was revealed that a vocabulary size of the most frequent 3,000 word families plus proper nouns and marginal words accounts for more than 95% lexical coverage of the lectures in the *Introduction to Computer Science* lectures (see Table 2). However, to achieve 98% lexical coverage, knowledge of the most common 5,000 word families plus proper nouns and marginal words is required. At the same time, lexical demand demonstrated significant variation across individual lectures. Specifically, to reach 98% lexical coverage^3^, one lecture required a lexical demand of the most common 3,000 word families, nine lectures required a lexical demand of 4,000 word families, eight lectures required a lexical demand of 5,000 word families, eight lectures required a lexical demand of 6,000 word families, one lecture required a lexical demand of 9,000 word families, and finally, one lecture required a lexical demand of 10,000 word families. Overall, results indicate a tremendous difference in lexical demand across individual lectures.

**TABLE 2:** Lexical coverage for the entire present corpus (%) with and without proper nouns and marginal words.

Word list	Coverage at each 1,000-word level without proper nouns and marginal words	Cumulative coverage with proper nouns and marginal words	Cumulative coverage without proper nouns and marginal words
1,000	87.08	88.28	87.08
2,000	5.58	93.86	92.66
3,000	3.07	96.93^a^	95.73^a^
4,000	0.78	97.71	96.51
5,000	0.48	98.19^b^	96.99
6,000	0.39	98.58	97.38
7,000	0.17	98.75	97.55
8,000	0.17	98.92	97.72
9,000	0.09	99.01	97.81
10,000	0.1	99.11	97.91
11,000	0.13	99.24	98.04^b^
12,000	0.04	99.28	98.08
13,000	0.04	99.32	98.12
14,000	0.03	99.35	98.15
15,000	0	99.35	98.15
16,000	0.02	99.37	98.17
17,000	0.02	99.39	98.19
18,000	0.05	99.44	98.24
19,000	0	99.44	98.24
20,000	0	99.44	98.24
21,000	0	99.44	98.24
22,000	0.01	99.45	98.25
23,000	0.01	99.46	98.26
24,000	0	99.46	98.26
25,000	0	99.46	98.26
Proper nouns	0.56		
Marginal words	0.64		
Off-list words	0.19		
Tokens	264,400		

^a^ Reaching over 95% coverage.

^b^ Reaching over 98% coverage.

For off-list words, there were eight words whose plural forms were treated as separate words. For example, *pseudocode* and *pseudocodes* were considered as two distinct words by AntWordProfiler. We fixed this issue through manual combination of the range and frequency values of each variant of these off-list words. We removed two off-list words (*mathematic* and *storaging*) as they were slips of the tongue by the professor when he was delivering the lectures. In the end, we were left with 137 off-list words, of which are 52 acronyms, accounting for 37.95% of the entire off-list words. Finally, a closer look at the off-list words shows that the majority of the off-list words (e.g., *pseudocode* or *algorithmic*) come from the computer science field.

In answering RQ2, we extracted 385 potentially new words in the 5,000 + frequency level lists. We carefully went through the target words and removed two proper nouns (i.e., Carol and Victor) that were misclassified by AntWordProfiler. For off-list words, we excluded 52 acronyms from the 137 off-list words since these words are believed to pose a minimal learning burden for learners. In the end, we were left with 468 words that are potentially unknown to students listening to the present lectures. These words were then sent to the course professor and the language instructor to rate their difficulty level. An intraclass correlation test was run to examine inter-rater reliability of the two raters. The intraclass correlation was 0.435, indicating a poor correlation between the two raters (e.g., Koo and Li, 2016). The result was unsurprising given that the rating was conducted by raters of different backgrounds. One rater was the course professor, while the other was a language instructor at the language center at the university. In this regard, they would likely perceive the difficulty level of the words in a different manner despite the fact that they have many years of working with students at the university, and that they both have an advanced English language proficiency level (i.e., C2 +). However, it should be highlighted that we intentionally included raters from dissimilar backgrounds because this would allow for the feedback on the difficulty level of the words from multiple perspectives (see Ackermann and Chen, 2013 for a discussion on this issue). At the same time, the goal of the current study is to estimate the number of words that are potentially unknown to the students, and in our study, only words judged *definitely known to students* by both raters would be removed. In doing so, the reliability of the results was still guaranteed, while the goal of the study was achieved. In the end, 113 words were judged *definitely known to students* by the two raters, and thus, we were left with 355 words that were potentially unknown to students.

In answering RQ3, we further classified these 355 words into four frequency bands: Words that occurred (1) 1–4 times, (2) 5–9 times, (3) 10–14 times, and (4) at least 15 times. Results show that out of the 355 potentially unknown words, 233 words that occurred 1–4 times, 51 words that occurred 5–9 times, 19 words that occurred 10–14 times, and 52 words that occurred at least 15 times. From the results on frequency, we excluded 233 words that occurred 1 to 4 times from further analysis because it would be difficult for students to acquire words of very low frequency incidentally even if they were explained by the course professor. In the next stage, we read over all the other 122 remaining words in the present lectures to see whether they were explained by the course professor. We found that 19 words were not explained by the course professor. Accordingly, we excluded these words from further analysis. In the next step, we classified the 103 words that occurred at least 5 times and were explained by the course professor into two different range groups: Words with a range value of 1–5 and words with a range value of more than 5. It was revealed that out of the 103 words, 73 words have a range value of 1–5, and the 30 remaining words have a range value of more than 5. Overall, we conclude that out of the 355 potentially new words, students could acquire 103 new words incidentally. These are the words that occurred at least 5 times and were explained by the course professor. When the range factor is taken into consideration, only 30 out of the 355 new words stand a good chance of being acquired by students. These are the words that have the frequency and range value of at least 5 and were explained by the course professor.

## 5. Discussion

Our study showed that lexical knowledge of the most frequent 3,000 word families plus proper nouns and marginal words would allow for more than 95% coverage of the *Introduction to Computer Science* EMI lectures. To achieve 98% coverage, vocabulary knowledge of the most common 5,000 word families plus proper nouns and marginal words is required. Moreover, two lectures required knowledge of the most frequent 9,000 or 10,000 word families to ensure 98% lexical coverage. Most recently, Reynolds et al. (2022) found that to reach 98% coverage of the EMI lectures in an *Introduction to English Language Teaching* course, the lexical knowledge of the most common 4,000 word families plus proper nouns and marginal words is needed. There are several reasons for the differences in the proposed vocabulary sizes. First, the size of the current corpus is over twice as big as that of Reynolds et al. (2022), with 264,400 and 128,498 tokens, respectively. A bigger corpus would perhaps contain more advanced words, thus creating a heavier lexical demand. The second, and perhaps more important, reason is related to the nature of the two types of EMI lectures: One belongs to the branch of computing and engineering (i.e., *Computer Science*), while the other is in the field of education (i.e., *English Language Teaching*). Disciplinary differences can contribute to different lexical demands across EMI lectures. As Peng and Xie (2021) reveal in their meta-analysis of the effectiveness of EMI in China’s higher education, discipline is a significant moderator for students’ English language learning achievement. Disciplinary differences may demand different English proficiency, including vocabulary, from students. By comparing results on lexical coverage across different fields, we want to emphasize that to help students reasonably understand the EMI lectures in their respective fields, more studies on lexical profiling on EMI lectures for specific disciplines are needed. Otherwise, students might have an imprecise and unrealistic estimation of the required vocabulary size for the comprehension of lectures in their fields.

The present study also revealed significant variations in lexical coverage across individual lectures. For instance, to reach 98% lexical coverage, eight lectures required a lexical demand of the most frequent 6,000 word families plus proper nouns and marginal words, and one lecture required a vocabulary size of the most frequent 10,000 word families. Such a finding provides a valuable pedagogical implication in that the course instructor perhaps should pay attention to more lexically demanding lectures, and if possible, create activities that can improve students’ understanding of the lectures. For example, students can be asked to complete a short quiz on key concepts before class or provided with reading materials in advance.

The corpus analysis found 355 words potentially new to the students that attended the computer science lectures. While listening to the lectures could reasonably provide students an opportunity for exposure to new words relevant to their discipline, it is questionable whether first-year students will have adequate vocabulary knowledge to incidentally infer the meaning of all of them. This number of new words embedded in the EMI lectures also calls for the need to integrate lexical development into EMI course objectives, as students would need to spend extra time and effort in learning these additional words to comprehend the course content. Without extra support, it may be unrealistic to assume that students attending EMI lectures will be able to incidentally acquire all unknown vocabulary encountered (Fang and Xie, 2019). Thus, this result signals both the possibility for students to incidentally acquire new subject-based vocabulary through listening to EMI lectures and the possible challenges that these unknown words may present to learners with insufficient vocabulary knowledge to reach 98% coverage of known words occurring in the lectures.

Explanation of the words by the lecturer is one pedagogical technique that could help reduce the burden of too many unknown words appearing in the lectures, as suggested by other researchers (Tian and Macaro, 2012; Yang and Sun, 2013; Zhao and Macaro, 2016; Webb and Nation, 2017; Jin and Webb, 2020). Applying this criterion to the list of 355 potentially new words and considering that students are likely to only pay attention to new words that occur repeatedly (i.e., 5 times or more), we narrowed the list down to 103 words that would likely be learned by students attending the *Introduction to Computer Science* lectures. However, even if the words are mentioned several times within a single lecture but are not heard again by students in other lectures might give the signal that such words are not important. It is also less likely that the students would engage with these words again at a later date unless prompted by the lecturer. Thus, further narrowing down this list of 103 words to those that occurred in at least five lectures resulted in only 30 words likely to have been learned incidentally from the listening to 40 h and 36 min of *Introduction to Computer Science* EMI lectures. On average, that equals less than one new word for every hour of listening to lectures.

The potential number of words to be incidentally acquired is rather small considering the time spent listening to the lectures. However, these results were mainly gained through assumptions that the students would need at least five encounters across five lectures and with at least one explicit explanation given by the lecturer. While it appears that the lecturer placed considerable emphasis on explanation of terms (103 of the words that occurred at a frequency of 5 or higher were given explicit explanations), he did not appear to have felt the need to recycle the new words introduced to the students, resulting in a limited range. It is likely that he did feel that students were encountering the words for the first time, or he would have been less inclined to have supplied explanations of their meanings. Since English vocabulary enhancement is not part of the assessment criteria and curriculum objectives of content courses, EMI lecturers might spend less time on vocabulary recycling throughout the EMI lectures (Fang and Xie, 2019; Galloway and Ruegg, 2020; Galloway and Rose, 2021). One unexpected finding of our study is the mismatch in the evaluation of the difficulty level of the words between the EMI lecturer of the computer science course and the language teacher from the same university, as indicated by the poor inter-rater reliability of 0.43. In particular, the EMI lecturer and the language teacher perceived word difficulty very differently even though they have extensive teaching experience at the same university. This mismatch may be caused by their varied educational backgrounds, disciplinary differences, and their understanding of student needs found in EAP and EMI classrooms.

## 6. Conclusion and implications

The analysis of the 28 computer science lectures has uncovered several findings related to the potential of incidentally acquiring vocabulary from listening. To reach over 95% lexical coverage, students would need to have acquired 3,000 word families plus proper nouns and marginal words. To reach 98% lexical coverage, the word family knowledge would need to have reached 5,000. While we found 355 potential words for incidental learning, after considering their frequency, range, and the lecturer’s explanation (or lack thereof), we only felt confident that 30 new words may potentially be acquired by students after listening to the EMI lectures.

There may be a need for EMI lecturers to receive EMI training to enhance pedagogical skills to provide linguistic support to students enrolled in their EMI courses, because the vocabulary knowledge development that occurs incidentally through listening to EMI lectures may be limited if the students’ language proficiency is limited (Wang et al., 2018; Galloway et al., 2020). If not possible, then collaborations with language instructors or linguistics experts might be a possibility as well (Lo, 2020).

Our finding regarding the mismatch in the evaluation of the difficulty level of the words between the EMI lecturer of the computer science course and the language teacher from the same university calls for the urgent need for the rethinking of EAP curriculum development when it comes to the EMI provision at the university. For instance, findings on the lexical coverage of academic lectures across different fields (e.g., Computer Science and Education) should be used to inform the lexical knowledge covered in the EAP curriculum to ensure that students participating in the EMI programs will be equipped with necessary lexical knowledge to boost their readiness for studying the EMI courses. At the same time, opportunities for collaboration and communication between the EMI lecturers and the language teachers are needed. For example, workshops and seminars on lexical coverage across various fields can be organized to provide the EMI lecturers and the language teachers a chance to share their perspectives on lexical demand expected in language courses compared with what is expected in content courses. EMI lecturers and EAP teachers are advised to co-construct and co-develop their curricula based on students’ contextualized needs. From there, suitable strategies and sustainable learning materials can be developed to support students’ vocabulary learning for a successful EMI learning experience.

Since we also found marked differences among the lexical demands across individual lectures, we suggest EMI lecturers consider providing students with vocabulary lists for the especially lexically demanding lectures. It may as well be crucial for the school administrators to reconsider the current admissions requirements using only English language proficiency tests. While these might work as a useful initial gauge, other tests such as the Vocabulary Levels Test (Webb et al., 2017) could be used to determine which level of vocabulary that learners should be aiming to master to become better prepared to engage with content knowledge materials. Professional development training for EMI lecturers that can explain the relationship between vocabulary size tests and the lexical coverage of lectures could be administered to provide EMI lecturers with the knowledge to assess their learners and provide proper support for lexically weak students.

While we found the lecturer devoted a great deal of time to explaining many of the 355 potentially unknown words, few of them were recycled throughout the lectures. We suggest that EMI lecturers should consider finding some ways to allow learners to revisit the new words introduced in their lectures. This could occur by explicitly incorporating or using the words in more lectures or it can be by asking the students to review class materials outside of class. In fact, it is likely that the lecturer that gave these lectures had some amount of out-of-class material that students worked with. However, it can only be guaranteed that students are exposed to the language that is provided through in-class materials.

## 7. Limitations and future directions

Although the findings are valuable, there are limitations of our study that require attention before generalizing our findings to other contexts. First, the corpus was composed of lectures given by a single lecturer for a single computer science course at a single university in Macau. It is difficult for us to draw implications for other contexts outside Macau. Range was examined as the number of lectures in which potentially unknown words occurred. We also admit that other contextual variables such as contextual richness and contextual diversity can affect incidental word gains. However, it is more practical to investigate these variables in well-controlled experimental or quasi-experimental studies. While our analysis was limited to the audio input from the lectures, students would be exposed to multimodal input as the lectures are accompanied with visual input through presentation slides. Although not analyzed in the current study, we admit that print and other visual exposure may change the learning dynamics of the input (Pellicer-Sánchez, 2022).

Although there was limited interaction between the lecturer and the students, future research may also consider how other learning activities such as question and answer sessions, discussion, and debate, among others, might affect incidental word learning outcomes. Future experimental studies could investigate these issues by comparing the incidental word gains of students that listen to the lectures, to those that view the lectures, and those that listen, view, and engage in other learning activities. In addition, to strengthen our findings, corpus analyses of lectures given by other lecturers within and beyond the field of computer science are crucial. Accordingly, we propose that future studies should focus on the EMI practices at the microlevel among broader disciplines and courses in addition to computer science. It will be beneficial for future studies to involve longitudinal classroom data to understand the effectiveness of EMI lectures for students’ incidental vocabulary acquisition. Meanwhile, it is essential to hear the voices of stakeholders from micro-, meso- and macro-levels, including policymakers, school administrators, government, and especially EMI lecturers. Most importantly, future studies may also benefit from the exploration of students’ perspectives while conducting similar corpus analyses like ours. It is also hoped that cross-discipline studies can be conducted to unpack the contextual challenges and benefits of the collaboration between EMI lecturers and EAP teachers.

## Data availability statement

The original contributions presented in this study are included in the article/Supplementary material, further inquiries can be directed to the corresponding author.

## Ethics statement

This study involving human participants was reviewed and approved by Sub-Panel on Social Science and Humanities Research at the University of Macau. Written informed consent to participate in this study was provided by the participants.

## Author contributions

BR contributed to the conceptualization, methodology, data collection, transcription, resources, supervision, project administration, funding acquisition, and in charge of review and editing. QP conducted formal analysis and was validated by BR, proofed the manuscript, and in charge of data curation and software. All authors wrote the first draft of the manuscript, contributed to manuscript revision during the review process, read, and approved the submitted version.

## Footnotes

1 We converted IELTS and TOEFL-iBT scores into CEFR levels as an extensive database search did not reveal any existing studies that relate vocabulary size to the IELTS and TOEFL-iBT scores.

2 Of note is the heated debate regarding the choice of a counting unit over whether it should be word family, lemma, or flemma [see the critical reviews in Cambridge University Press (2021)]. It is still inconclusive if there is superiority of any one of the counting units. In our study, we employed word family as the counting unit because we wanted to follow practices discussed in previous studies on incidental vocabulary learning through listening (e.g., Chen and Teng, 2017; Pavia et al., 2019; Nie et al., 2022). By doing so, the present findings can be comparable across prior studies.

3 The lexical demand of individual lectures is included in the Supplementary material.
